# Neither myonuclear accretion nor a myonuclear domain size ceiling is a feature of the attenuated hypertrophic potential of aged human skeletal muscle

**DOI:** 10.1007/s11357-022-00651-y

**Published:** 2022-09-09

**Authors:** Matthew S. Brook, Daniel J. Wilkinson, Janelle Tarum, Kyle W. Mitchell, Jonathan L. Lund, Bethan E. Phillips, Nathaniel J. Szewczyk, Fawzi Kadi, Paul L. Greenhaff, Ken Smith, Philip J. Atherton

**Affiliations:** 1 MRC-Versus Arthritis Centre for Musculoskeletal Ageing Research and NIHR Nottingham BRC, Centre of Metabolism, Ageing and Physiology (COMAP), School of Medicine, University of Nottingham, Derby, UK; 2 School of Life Sciences, University of Nottingham, Nottingham, UK; 3 School of Health Sciences, Örebro University, Örebro, Sweden

**Keywords:** DNA synthesis, Skeletal muscle, Ageing, Resistance exercise, Myonuclei

## Abstract

Ageing limits growth capacity of skeletal muscle (e.g. in response to resistance exercise), but the role of satellite cell (SC) function in driving this phenomenon is poorly defined. Younger (Y) (~ 23 years) and older (O) men (~ 69 years) (normal-weight BMI) underwent 6 weeks of unilateral resistance exercise training (RET). Muscle biopsies were taken at baseline and after 3-/6-week training. We determined muscle size by fibre CSA (and type), SC number, myonuclei counts and DNA synthesis (via D_2_O ingestion). At baseline, there were no significant differences in fibre areas between Y and O. RET increased type I fibre area in Y from baseline at both 3 weeks and 6 weeks (baseline: 4509 ± 534 µm^2^, 3 weeks; 5497 ± 510 µm^2^ *P* < 0.05, 6 weeks; 5402 ± 352 µm^2^ *P* < 0.05), whilst O increased from baseline at 6 weeks only (baseline 5120 ± 403 µm^2^, 3 weeks; 5606 ± 620 µm^2^, 6 weeks; 6017 ± 482 µm^2^ *P* < 0.05). However, type II fibre area increased from baseline in Y at both 3 weeks and 6 weeks (baseline: 4949 ± 459 µm^2^, 3 weeks; 6145 ± 484 µm^2^ (*P* < 0.01), 6 weeks; 5992 ± 491 µm^2^ (*P* < 0.01), whilst O showed no change (baseline 5210 ± 410 µm^2^, 3 weeks; 5356 ± 535 µm^2^ (*P* = 0.9), 6 weeks; 5857 ± 478 µm^2^ (*P* = 0.1). At baseline, there were no differences in fibre myonuclei number between Y and O. RET increased type I fibre myonuclei number from baseline in both Y and O at 3 weeks and 6 weeks with RET (younger: baseline 2.47 ± 0.16, 3 weeks; 3.19 ± 0.16 (*P* < 0.001), 6 weeks; 3.70 ± 0.29 (*P* < 0.0001); older: baseline 2.29 ± 0.09, 3 weeks; 3.01 ± 0.09 (*P* < 0.001), 6 weeks; 3.65 ± 0.18 (*P* < 0.0001)). Similarly, type II fibre myonuclei number increased from baseline in both Y and O at 3 weeks and 6 weeks (younger: baseline 2.49 ± 0.14, 3 weeks; 3.31 ± 0.21 (*P* < 0.001), 6 weeks; 3.86 ± 0.29 (*P* < 0.0001); older: baseline 2.43 ± 0.12, 3 weeks; 3.37 ± 0.12 (*P* < 0.001), 6 weeks; 3.81 ± 0.15 (*P* < 0.0001)). DNA synthesis rates %.d^−1^ exhibited a main effect of training but no age discrimination. Declines in myonuclei addition do not underlie impaired muscle growth capacity in older humans, supporting ribosomal and proteostasis impairments as we have previously reported.

## Introduction

Ageing is associated with incipient declines in skeletal muscle mass, diminished muscle function and frailty—a syndrome known as sarcopenia [1]. The decline in muscle bulk, force-producing capacity and motor control with ageing is the result of neurodegenerative processes coupled to loss of cell mass [2, 3]. These processes are stimulated by a general loss of homeostasis and to some extent, behavioural changes favouring net muscle catabolism [4]. These include the loss, and expansion, of motor units [5]; anabolic resistance to key homeostatic environmental cues regulating habitual muscle mass (e.g. food and movement [6–8]); and also, general reductions in habitual activity associated with ageing [9]. Given the burgeoning of individuals living into very old age, albeit with more chronic disease burden and polypharmacy, there is a shift towards focussing upon health, rather than lifespan.

Despite heavy investment in alternative therapies, the most potent stimulation for the improvement in muscle mass and function remains resistance exercise training (RET). Nonetheless, while resistance exercise is robustly able to improve muscle function in older age [10, 11], it is clear there exist age-related deficits in hypertrophic adaptations to full-supervised RET [12] which act to limit the utility of RET as a countermeasure to sarcopenia. Indeed, we have shown that both longer term (20 weeks) [13] and shorter term RET regimens (6 weeks) [8] fail to stimulate muscle mass accretion robustly in cohorts of older individuals, in comparison to younger cohorts, albeit functional benefits are attainable. As such, there is a need to determine the mechanisms underpinning age-related adaptive deficits in muscle hypertrophy.

Skeletal muscle mass is controlled by the balance in muscle protein synthesis (MPS) and muscle protein breakdown (MPB) [14]. In response to RET, individual bouts of exercise transiently stimulate MPS (assuming intake of adequate protein nutrition) through integrated contractile, nutritional, hormonal and mechano-signalling [7, 14, 15] leading to net increases in absolute synthesis rates over days-to-weeks and thus protein accretion and eventual hypertrophy [16]. A second key player in muscle deposition is ribosomal biogenesis, which makes sense teleologically, since sustained increases in MPS require an increase in the rate of mRNA translation to generate greater copy numbers of template proteins. Evidence of a role for upregulation of rRNA being important for muscle growth arises from previous work demonstrating acute upregulation of rRNA species [17, 18] and total RNA ([19]—of which ~  > 85% in rRNA), in addition to the total RNA pool correlating to muscle mass gains [8, 17, 20]. Moreover, we previously showed that long-term MPS and RNA synthesis correlated closely [21], and upregulation of MPS and ribosomal biogenesis was blunted in older individuals undertaking an RET programme [8]. These data suggest RNA and MPS deficits at least partially underlie age-related deficits in anabolic responses to RET, which are perhaps co-regulated.

The final major area of control over muscle mass is that of satellite cells (SC)—muscle stem cells (non-post-mitotic) capable of mitosis and donation of daughter cells into sub-sarcolemma nuclei [22]. Originally shown to be required for muscle regeneration following trauma/myotoxin challenge [23, 24], their role in regulating muscle hypertrophy (the myonuclear domain hypothesis [25, 26]) in response to loading paradigms has been hotly contentious, e.g. with genetic models of PAX7 + depletion coupled to synergist ablation suggesting a role [27] or no major role [28] in muscle hypertrophy. In human trials, others have shown that ‘high responders’ demonstrate a greater number of myonuclei and accrue more myonuclei with RET, suggesting a role in muscle growth [29]. Nonetheless, this issue has received little attention in relation to ageing; that said, earlier work from Bamman’s lab suggested impaired age (and gender, in younger women) related inductions in myonuclei number, using NCAM + staining approaches, which may inadequately represent the heterogeneous SC pool (i.e. vs. PAX7) [26]. Furthermore, pre-clinical work by Peterson’s lab has suggested that age-related anabolic resistance in mice is unaffected by genetic SC depletion and therefore that failed muscle growth is unaffected by SC loss—suggesting other factors predominate in muscle mass regulation [30]. We have previously reported diminished muscle hypertrophic potential in older individuals [8]. In the present study, we sought to further clarify the (likely) role of SC in this group of older humans [8] in regulating the pre-established age-related deficits in muscle hypertrophic responses to RET.

## Methods

### Subject characteristics and ethics

Ten healthy younger (23 ± 1 years, BMI: 24 ± 1 kg/m^2^) and older (69 ± 1 years, BMI 25.8 ± 1 kg/m^2^) men were recruited. Volunteers were screened by medical questionnaire, physical examination and resting electrocardiogram, with exclusions for metabolic, respiratory and cardiovascular disorders or any other symptoms of ill health. Subjects had clinically normal blood chemistry, were normotensive (< 140/90) and were not prescribed any medications: all subjects performed activities of daily living and recreation but did not undertake any RET other than that described in the study and had not participated in any RET within the last 12 months. All subjects provided their written, informed consent to participate after all procedures and risks (in relation to muscle biopsies, blood sampling, etc.) were explained. This study was approved by the University of Nottingham Ethics Committee, with all studies conducted according to the declaration of Helsinki and preregistered (clinicaltrials.gov registration no. NCT02152839).

### Conduct of the study

The conduct of the study has been described in detail previously [8]. In brief, this study involved a RET program over a 6‐week period. On the first day of study, subjects arrived at the laboratory at 08.30 h following an overnight fast, and subjects completed the first session of RET consisting of unilateral knee extension exercise (i.e. 6 × 8 repetitions at 75% of one repetition maximum (1‐RM)). Bilateral biopsies were taken from the vastus lateralis (VL) muscle 60–90 min (75 ± 2 min) after unilateral exercise under sterile conditions, using the conchotome biopsy technique (Dietrichson et al. 1987) with 1% lidocaine (B. Braun, Melsungen, Germany) as local anaesthetic. Muscle was rapidly dissected free of fat and connective tissue, washed in ice‐cold phosphate‐buffered saline (PBS) and then frozen in liquid N_2_ and stored at − 80 °C until further analysis. Immediately post‐RET, subjects provided a saliva sample (collected in sterile plastic tubes) and consumed a 150-ml bolus of D_2_O (70 atom%; Sigma‐Aldrich, Poole, UK), with the aim to label the body water pool to ∼0.2% atom percent excess (APE), which was maintained in a pseudo‐steady state with weekly top‐up boluses (~ 50 ml week^−1^). In addition, venous blood samples were collected into lithium heparin–coated tubes, immediately cold-centrifuged at 1750 g, with plasma fractions aliquoted and frozen at − 80 °C until analysis. Thereafter, subjects returned to the lab 3 times per week to undertake supervised unilateral RET with 1‐RM assessments of the trained leg every ∼10 days to ensure progressive intensity. Further bilateral muscle biopsies (∼90 min after RET) were taken at 3 and 6 weeks. For the temporal monitoring of body water enrichment, each participant provided a saliva sample on RET visits > 60 min after their last meal or drink, with extra samples taken ∼3 h after weekly 50-ml boluses to ensure body water enrichment was accurately represented. These were collected in sterile plastic tubes and immediately cold‐centrifuged at 16,000 g to remove any debris that might be present; they were then aliquoted into 2-ml glass vials and stored frozen at − 20 °C until analysis.

### Immunohistochemical analysis

**Fig. 1:**
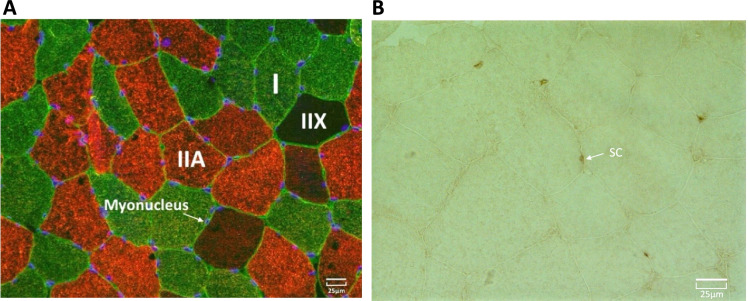
**A** Immunofluorescent staining of human *m. vastus lateralis* for myosin heavy chain (MHC) type I (green), MHC 2a (red), MHC IIX (unstained) and laminin (green). Immunostaining was coupled with DAPI to visualize nuclei (blue). The arrow indicates a myonucleus (scale bar = 25 μm). **B** Staining of human *m. vastus lateralis* against Pax7. The arrow indicates satellite cells visualized using DAB peroxidase kit (scale bar = 25 μm)

Muscle cross sections (younger = 8, older = 7 *(reduced n due to tissue limitations)*) were cut at − 20 °C with a cryostat microtome (Leica Biosystems, CM 1850). After 4-h air-drying at room temperature, sections were fixed with 2% paraformaldehyde and incubated in blocking buffer for 30 min. Satellite cells were stained using a mouse monoclonal antibody against Pax7 (Anti-PAX7 antibody [PAX7497] (ab199010)). Sections were then incubated with a biotinylated goat-anti-mouse secondary antibody (ab64255) followed by an incubation with Vectastain ABC reagent (Vector Laboratories, PK6100). Diaminobenzidine (DAB) Peroxidase Substrate Kit (Vector Laboratories, SK-4105) was used for the visualization of the antibody binding. Immunofluorescence was subsequently used for the labelling of the basal lamina of muscle fibres and the identification of muscle fibre types. The slides were incubated for 60 min at 37 °C with the primary antibodies against laminin (D18), MHC IIa (SC-71) and MHC I (BA-F8) (Developmental Studies Hybridoma Bank). Fluorescent labelled Alexa 488 and 568 (Alexa Fluor, Life Technologies) secondary antibodies were used. The slides were then mounted with ProLong Gold antifade reagent with DAPI for staining of myonuclei in blue (Life Technologies). Satellite cells were stained brown, type I muscle fibres were stained green, and type IIA stained red and type IIX were unstained. Following the staining protocol, muscle sections were imaged using fluorescence microscopy at 20 × magnification (Fig. 1). Fibre CSA was quantified using manual identification, where fibre circumference was outlined using Image J Software. Only fibres that displayed intact cell membranes without deformations and elongated shape were included the CSA analysis. The average number of type I fibres analysed was 98 ± 9, and type IIA fibres 161 ± 14, with total number of fibres being 260 ± 20. Type IIX fibres were not included in the analysis due to insufficient fibres meeting the inclusion criteria. To identify fibre type-specific satellite cell and myonuclei number, Pax7 co-staining with type I/II MyHC and laminin was used. Pax7 + /DAPI + nuclei residing within the laminin were counted as satellite cells and the DAPI + /Pax7- nuclei were counted as myonuclei.

### DNA extraction, digestion and derivatization

To extract DNA (younger = 10, older = 7 *(reduced n due to tissue limitations)*), approximately 20–30 mg of muscle was homogenized in extraction buffer (5 µl/mg) containing 0.1 M Tris·HCl, pH 8, 0.01 M EDTA, pH 8 and 1 M NaCl. Proteinase K was added to a final concentration of 50 µg/µl and placed at 55 °C for ~ 2 h with occasional mixing until complete digestion had occurred. To the extractions, an equal volume of phenol–chloroform-isoamyl alcohol (25:24:1) was added and inverted several times to mix, and the upper aqueous layer was removed to a clean Eppendorf after centrifugation at 13,000 rpm for 10 min. To remove additional protein, an equal volume of chloroform-isoamyl alcohol (24:1) was added to the aqueous layer and repeated as above. To precipitate DNA, an equal volume of isopropanol was added to the aqueous layer, inverted several times and centrifuged at 13,000 rpm for 20 min. The pellet was washed three times in 70% ethanol; air-dried; resuspended in 22 µl of molecular biology water; digested with 5 µl of 375 mM sodium acetate (pH 4.8) and 750 µM ZnSO_4_ containing 0.5 units of nuclease S1 and 0.25 units of potato acid phosphatase; and placed at 37 °C overnight. Hydrolysates were then reacted with 10 µl of *O*-benzylhydroxylamine (2% wt/vol) and 7.5 µl of acetic acid at 100 °C for 30 min. Samples were allowed to cool at room temperature before the addition of 10 µl of 1-methylimidazole and 100 µl of acetic anhydride. The reactions were transferred to a boiling tube and quenched by the addition of 2 ml of double-distilled water. The newly formed derivatives were extracted by the addition of 750 µl of dichloromethane (DCM) vortex mixed, and phases were allowed to separate. By prewetting the tip with DCM, the lower layer was removed to a clean boiling tube, and the procedure was repeated. DCM extracts were then dried and resuspended in 40 µl of ethyl acetate for GC–MS/MS analysis.

### GC–MS/MS instrument conditions and fractional synthesis rate calculation

To measure DNA enrichment, 2 µl of sample was injected into a TRACE 1310 Gas Chromatograph connected to TSQ 8000 triple quadrupole GC–MS/MS (Thermo Scientific). Samples were injected on splitless mode with inlet temperature at 280 °C. GC ramp conditions were 120 °C for 1 min, ramp to 280 °C at 10 °C/min and hold for 3 min. Selected reaction monitoring (SRM) was performed for the mass-to-charge ratios of 203.1–82.1 and 204.1–84.1 representing the M and M^+1^ ions with a collision-induced dissociation energy of 6 eV. Enrichment was calculated as M^+1^/(M + M^+1^) with the mole percent excess (MPE) expressed as difference from unlabelled D_2_O free samples. Fractional synthesis rates (FSR) were calculated as FSR (%/day) = (r-MPE)/[(p-MPE) × *t*] × 100, where r-MPE is the excess enrichment of bound deoxyribose, p-MPE is the mean precursor enrichment over the time period and *t* is the time between samples. The p-MPE was calculated as the average body water enrichment multiplied by number of labelled hydrogens of 5.6 as previously determined [31]. Samples were run in triplicate alongside standard curves of known deoxyribose standards, and the average of both peaks was used in the results. Additionally, unlabelled samples were injected in different quantities to determine signal size effects.

### Gene expression analysis

Total RNA (younger = 10, older = 10) was isolated by homogenizing 5–10 mg of muscle in 200 μl of TRizol (Life Technologies/Thermo Fisher Scientific) using two stainless steel beads (TissueLyser II, Qiagen, UK) for 1 min at 30 s^−1^. Samples were placed at ambient temperature for 10 min before 80 μl of chloroform was added and samples vortexed and incubated at ambient temperature for 10 min. After centrifugation at 12,000 g for 15 min at 4 °C, the upper aqueous layer was removed and RNA precipitated with an equal volume of isopropanol, incubation at room temperature for 10 min and subsequent centrifugation at 7000 g for 10 min at 4 °C. The pellet was washed twice with 1 ml of 80% ethanol, dissolved in 22 μl of RNA-free water and quantified by spectrophotometry (NanoDrop Lite). For RT-qPCR, 500 ng of total RNA was reversed-transcribed with the high-capacity cDNA reverse transcription kit (Life Technologies) according to the manufacturer’s protocol. Resulting cDNA was diluted 1:5 and 1 μl was added per well of 384-optical well plates (Life Technologies). Exon specific primers were mixed with SYBR Select Master Mix (Life Technologies) and 11 μl of master mix was added to each well, with samples run in triplicate. Primer sequences used were myogenin, Forward *5′‐CCAGGGGATCATCTGCTCACG‐3′*, Reverse *5′‐GGTTTCATCTGGGAAGGCCA‐3*′, and MyoD, Forward *5′‐CTCCGACGGCATGATGGACTA‐3′*, Reverse *5′‐TGGGCGCCTCGTTGTAGTA‐3′*. Thermal cycling conditions were 2 min at 50 °C followed by 2 min at 95 °C and 40 cycles of 15 s at 95 °C and 60 s at 60 °C on a ViiATM 7 Real-Time PCR System (Life Technologies). To control for RNA input, peptidylprolyl isomerase A levels were measured, and target mRNA expression was quantified using the ∆∆Ct method (Schmittgen & Livak, 2008).

### Statistical analysis

Descriptive statistics were produced for all data sets to check for normal distribution (accepted if *P* > 0.05) using a Kolmogorov–Smirnov test. All data are presented as means ± SEM or as a boxplots where the whiskers show the maximum and minimum, boxes represent the interquartile range, the cross indicates the mean and the horizontal line the median. All data sets were analysed by repeated-measures two-way ANOVA with a Bonferroni correction using GraphPad Prism 5 software (La Jolla, CA). Correlations were assessed using Pearson product moment correlation coefficient. The α-level of significance was set at *P* < 0.05.

## Results

### VL fibre area, myonuclei number, myonuclear domain and SC number

**Fig. 2:**
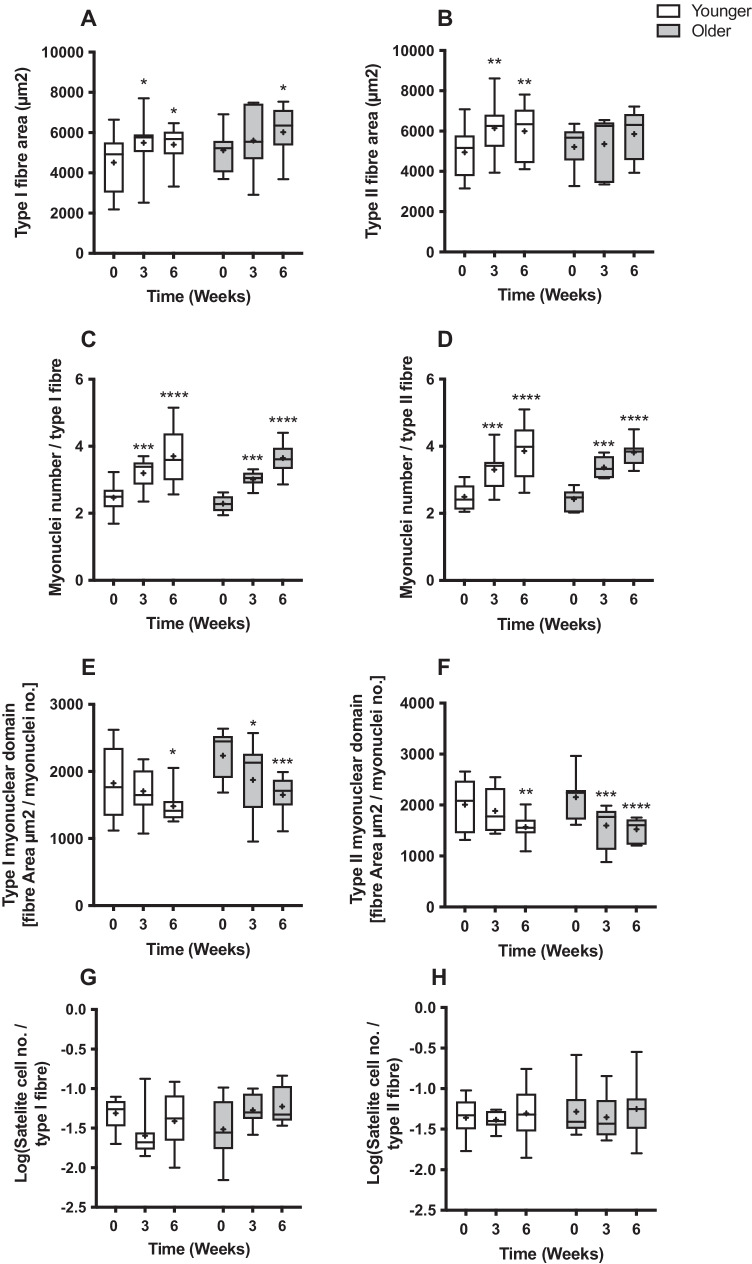
The effect of 6-week progressive RET on fibre area in **A** type I and **B** type II fibres, myonuclei number per **C** type I and **D** type II fibres, myonuclear domain in **E** type I and **F** type II fibres and Log(satellite cell number) per **G** type I and **H** type II fibres. Significantly different from baseline *P < 0.05, **P < 0.01, ***P < 0.001, ****P < 0.0001

At baseline, there were no significant differences in fibre area between Y and O. With RET, type I fibre area showed a main effect of time (*P* < 0.01), with increases from baseline in Y at both 3 weeks and 6 weeks (baseline: 4509 ± 534 µm^2^, 3 weeks; 5497 ± 510 µm^2^ *P* < 0.05, 6 weeks; 5402 ± 352 µm^2^ *P* < 0.05), with O showing an increase from baseline at 6 weeks only (baseline 5120 ± 403 µm^2^, 3 weeks; 5606 ± 620 µm^2^, 6 weeks; 6017 ± 482 µm^2^ *P* < 0.05) (Fig. 2A), with no group or interaction effects. There was a main effect of time (*P* < 0.01) for type II fibre area; however, increases occurred from baseline in Y at both 3 weeks and 6 weeks (baseline: 4949 ± 459 µm^2^, 3 weeks; 6145 ± 484 µm^2^ (*P* < 0.01), 6 weeks; 5992 ± 491 µm^2^ (*P* < 0.01)), whilst O showed no change (baseline 5210 ± 410 µm^2^, 3 weeks; 5356 ± 535 µm^2^, 6 weeks; 5857 ± 478 µm^2^) (Fig. 2B), with no group or interaction effects.

At baseline, there were no significant differences in fibre myonuclei number, or myonuclear domain size between Y and O. With RET, there was a main effect of time (*P* < 0.0001) for type I fibre myonuclei number, showing increases from baseline in both Y and O at 3 weeks and 6 weeks with RET (younger: baseline 2.47 ± 0.16, 3 weeks; 3.19 ± 0.16 (*P* < 0.001), 6 weeks; 3.70 ± 0.29 (*P* < 0.0001); older: baseline 2.29 ± 0.09, 3 weeks; 3.01 ± 0.09 (*P* < 0.001), 6 weeks; 3.65 ± 0.18 (*P* < 0.0001)) (Fig. 2C) with no group or interaction effects. Similarly, there was a main effect of time (*P* < 0.0001) for type II fibre myonuclei number, increasing from baseline in both Y and O at 3 weeks and 6 weeks (younger: baseline 2.49 ± 0.14, 3 weeks; 3.31 ± 0.21 (*P* < 0.001), 6 weeks; 3.86 ± 0.29 (*P* < 0.0001); older: baseline 2.43 ± 0.12, 3 weeks; 3.37 ± 0.12 (*P* < 0.001), 6 weeks; 3.81 ± 0.15 (*P* < 0.0001)) (Fig. 2D) with no group or interaction effects.

Myonuclear domain size in type I fibres showed a main effect of time (*P* < 0.0001) with RET, with no change with at 3 weeks in Y (baseline: 1826 ± 195 µm^2^, 3 weeks; 1705 ± 127 µm^2^) yet a decrease at 6 weeks (1483 ± 91 µm^2^ (*P* < 0.05)). O showed a decrease at both 3 and 6 weeks (baseline 2236 ± 141 µm^2^, 3 weeks; 1873 ± 212 µm^2^ (*P* < 0.05), 6 weeks; 1650 ± 109 µm^2^ (*P* < 0.001)) (Fig. 2E) with no group or interaction effects. Similarly, in type II fibres, myonuclear domain size showed a main effect of time (*P* < 0.0001) with RET, with no change at 3 weeks in Y (baseline: 2010 ± 189 µm^2^, 3 weeks; 1884 ± 149 µm^2^) yet a decrease at 6 weeks (1568 ± 94 µm^2^ (*P* < 0.01)). O showed a decrease at both 3 and 6 weeks (baseline 2153 ± 170 µm^2^, 3 weeks; 1598 ± 160 µm^2^ (*P* < 0.001), 6 weeks; 1525 ± 85 µm^2^ (*P* < 0.0001)) (Fig. 2F) with no group or interaction effects.

At baseline, there were no significant differences in SC number per fibre between Y and O. With RET, there were no significant main effects for type I fibre SC number (younger: baseline 0.054 ± 0.007, 3 weeks; 0.036 ± 0.016, 6 weeks; 0.050 ± 0.013 [Log(younger: baseline − 1.31 ± 0.07, 3 weeks; − 1.59 ± 0.13, 6 weeks; − 1.41 ± 0.12)]; older: baseline 0.042 ± 0.012, 3 weeks; 0.059 ± 0.010, 6 weeks; 0.069 ± 0.015) [Log(older: baseline − 1.52 ± 0.14, 3 weeks; − 1.27 ± 0.07, 6 weeks; − 1.23 ± 0.08)]) (Fig. 2G). Similarly, there were no significant main effects for type II fibre SC number (younger: baseline 0.049 ± 0.008, 3 weeks; 0.043 ± 0.004, 6 weeks; 0.063 ± 0.016, [Log(younger: baseline − 1.36 ± 0.08, 3 weeks; − 1.38 ± 0.04, 6 weeks; − 1.31 ± 0.11)]; older: baseline 0.070 ± 0.025, 3 weeks; 0.053 ± 0.013, 6 weeks; 0.077 ± 0.027 [Log(older: baseline − 1.28 ± 0.10, 3 weeks; − 1.35 ± 0.09, 6 weeks; − 1.25 ± 0.12)]) (Fig. 2H).

### VL DNA synthesis rate and mRNA expression

**Fig. 3:**
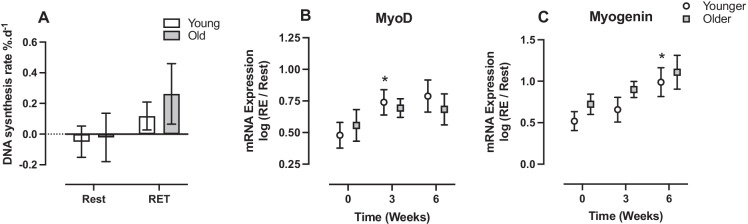
The effect of 6-week progressive RET on **A** DNA synthesis rate and acute mRNA expression of **B** MyoD and **C** myogenin. Significantly different from baseline *P < 0.05

At rest, there was no difference in VL DNA synthesis rate between Y and O over 6 weeks (younger: − 0.05 ± 0.10%.d^−1^, older: − 0.02 ± 0.16%.d^−1^), and there was a main effect of time (*P* < 0.05) with RET on VL DNA synthesis (younger: 0.12 ± 0.09%.d^−1^, older: 0.26 ± 0.19%.d^−1^) (Fig. 3A) with no group or interaction effects. At baseline, there was no difference in the MyoD mRNA expression 60–90 min after a bout of RE in Y or O. Similarly, over 6 weeks of RET, there were no significant main effects (younger: baseline 0.48 ± 0.10, 3 weeks; 0.74 ± 0.10, 6 weeks; 0.78 ± 0.13; older: baseline 0.56 ± 0.12, 3 weeks; 0.69 ± 0.07, 6 weeks; 0.68 ± 0.12) (Fig. 3B). There was no difference in the myogenin mRNA expression 60–90 min after a bout of RE in Y or O. Over 6 weeks of RET, there was a main effect of time (*P* < 0.05), showing an increase in Y only at 6 weeks (younger: baseline 0.52 ± 0.11, 3 weeks; 0.65 ± 0.15, 6 weeks; 0.99 ± 0.17 (*P* < 0.05); older: baseline 0.72 ± 0.12, 3 weeks; 0.90 ± 0.09, 6 weeks; 1.11 ± 0.20) (Fig. 3C).

### Correlations

**Fig. 4:**
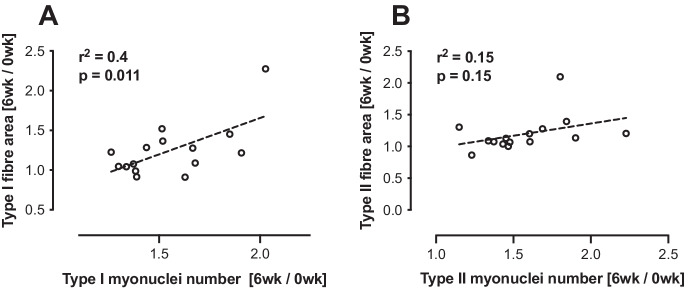
Correlations between the change in myonuclei number vs change in fibre area for **A** type I fibres and **B** type II fibres

The change in fibre area 0–6 weeks vs. the change in myonuclear number 0–6 weeks showed a significant positive correlation in type I fibres only (*P* = 0.011, *r*^2^ = 0.4) (Fig. 4A) and not type II fibres (*P* = 0.15, *r*^2^ = 0.15) (Fig. 4B).

## Discussion

The role of satellite cell–mediated myonuclear addition in skeletal muscle hypertrophic adaptations remains contentious, despite a wealth of both animal and human studies [26–28, 32, 33]. Overall, in the current study, we did not find any significant interaction effects, with both younger and older individuals increasing myonuclear number, highlighting no impairment in satellite cell activation and addition.

We have previously reported that muscle mass gains in these older individuals were markedly blunted at the level of whole muscle(s) [8]. The present work confirms this at the level of individual muscle fibres, particularly in relation to type II fibres, where we observed no detectable increase in myofibre CSA in older individuals following 6-week RET, as opposed to the increase observed in younger muscles both at 3 and 6 weeks of RET. In our prior work [8], we showed that blunted muscle growth with age was reflected in both attenuated MPS and ribosomal biogenesis, predominantly reflecting rRNA as the largest RNA pool contributor. These findings were a catalyst for our continuation to investigate facets relating to DNA/SC activity in muscle and their possible involvement in the anabolic resistance observed in older muscle.

Overall, whether, and the degree to which, myonuclear accretion is required to support the rising transcriptional demands of muscle hypertrophy remains unclear. The addition of new myonuclei can only be achieved through the coordinated activation, proliferation and fusion of muscle satellite cells with existing fibres [34, 35]. Nonetheless, even genetic models of e.g. PAX7 + manipulation have shown variable results as to the ‘requirement’ of muscle SC for hypertrophy (the reader is referred to the following literature review on this topic [36]). Nonetheless, this as a physiological question poses further complexity. In humans, researchers have demonstrated an increase in satellite cell pool size in response to both acute [37] and long-term RET [33], whilst muscle fibre hypertrophy is often accompanied with increased myonuclear number, correlating with muscle growth and being observed in both men and women [26]. In the present study, alongside hypertrophy, we observed an increase in type I/II fibre myonuclei number in younger men after just 3 weeks which was also evident after 6 weeks. These data are generally in-keeping with the notion that myonuclear addition is a common response of resistance exercise training exposure, although whether correlating or not, this does not provide irrepressible evidence of a mechanistic role, e.g. if SC activity were alternatively driven through mechanisms independent of proteostasis, for instance.

The benefit of simultaneous and allied investigation of a younger vs. older group, as herein, is that it enables comparisons to be drawn under the distinctly predictable scenarios (unilateral RET) in an established model of *successful* vs. *failed* muscle growth [8]. It was notable in our older men that despite only modest hypertrophy seen in type I fibres only, myonuclei accretion (as the end result of new nuclei influx from SC) was well preserved in both type I and II fibres. This is in line with others’ studies in individuals of a similar age [33, 38], but also perhaps explicably in contrast to results in very old (octogenarians) humans [32]. In terms of links between fibre size and myonuclear accretion, in a regimen of training-detraining-retraining in older individuals [39], myonuclear number tended to follow individual changes in type II fibre size. In contrast, earlier work from Petrella et al. [26] suggested that myonuclear addition was specific to younger individuals, and did not occur in older people (~ 63 years). The reason for this latter discrepancy is unclear; the training mode (knee extensor) was similar and the training period was longer and the study *N* larger than here, but similar in size and duration to other trials illustrating robust increases in myonuclei in older age [38]. Finally, disparate links have been shown between fibre CSA and myonuclei number. For instance, RET-induced fibre hypertrophy in younger individuals occurred with no change in myonuclei content [40], and despite fibre atrophy with ageing, myonuclear number was shown to be higher [41]. It is possible that conflicting literature is a result of differing methodological approaches influencing data. Nonetheless, our work supports the thesis of impaired hypertrophy (i.e. anabolic resistance) being separated from myonuclear accretion, with this being reflected in a markedly reduced myonuclear domain size.

Finally, albeit in a small sample size and low turnover pool (and one of mixed fibre population), we investigated DNA synthesis rates in muscle biopsies. Others’ have also investigated DNA synthesis in human skeletal muscle using the D_2_O approach and have shown increased turnover of this pool [42]. In response to aerobic exercise training, Robinson et al. suggest it is possible that the proportion of newly synthesized DNA could be a result of satellite cell ‘turnover’ [42]. However, caution must be taken when interpreting these results as there are many other resident cell types within muscle that could contribute to new DNA synthesis including fibroblasts, macrophages and endothelial cells [43]. Over 6 weeks, we could not detect active DNA synthesis in muscle at rest, indicating minimal proliferation of resident cells, yet, we report a main effect of resistance training across both age groups. Whilst many cell types may be activated by resistance training, myonuclei represent the largest proportion of nuclei within muscle [44]. While far from definitive, an increase in myonuclei number and overall DNA synthesis may point to a maintenance of signals driving SC activity (and ensuing myonuclear addition) in older age. However, further work isolating specific cell types prior to DNA synthesis analysis is required to confirm this. Due to tissue and RNA limitations due to our previous work with these samples, of the myogenic regulatory factors involved in satellite cell activation, we were only able to determine MyoD and myogenin gene expression in the present trial and noted small but statistically different within group differences, i.e. an induction in younger but not older individuals. While in agreement with our prior work showing a generalized blunting of anabolic programming with age [45], these measures lack rigour in relation to our major end-points of myonuclear addition and muscle fibre hypertrophy. Further work is needed to determine links between DNA synthesis and satellite cell/myonuclei outcomes, although our preliminary data illustrating a generalized effect of exercise is in line with others [42] and is not in disagreement with findings on myonuclear accretion.

While this remains a contentious area, our study comparing younger and older humans (albeit men only) supports the notion that myonuclei accretion as an ‘end-product’ of SC activity is not impaired in older muscle, despite the failure to observe muscle hypertrophy following 6-week RET. Along with our previous findings, we suggest that proteostasis (protein turnover and ribosomal content) is more likely to underlie the phenomenon of anabolic resistance, which may also imply the important role of distinct upstream pathways. While age, sex, fitness/health and protocol of study may influence outcomes from such human studies, we contend that myonuclear accretion is not rate limiting for physiological muscle hypertrophy to RET in older humans.
